# Retrospective study of bovine besnoitiosis in the Auvergne Rhône-Alpes region in France

**DOI:** 10.3389/fvets.2025.1621589

**Published:** 2025-08-21

**Authors:** Tiphaine Lahondes, Nuno Carolino, Sérgio Ramalho Sousa, Helga Waap, Elisabete Gomes Martins

**Affiliations:** ^1^Vasco da Gama Research Center/Vasco da Gama University School, Coimbra, Portugal; ^2^Instituto Nacional de Investigação Agrária e Veterinária, I.P.-Unidade Estratégica de Investigação e Serviços de Biotecnologia e Recursos Genéticos, Estacão Zootécnica Nacional – Polo de Investigação da Fonte Boa, Vale de Santarém, Portugal; ^3^Centre for Interdisciplinary Research in Animal Health (CIISA) and AL4Animals, Faculdade de Medicina Veterinária, Universidade de Lisboa, Lisboa, Portugal; ^4^Laboratório de Parasitologia, Instituto Nacional de Investigação Agrária e Veterinária, Oeiras, Portugal

**Keywords:** *Besnoitia besnoiti*, dairy cows, France, prevalence, Auvergne Rhône-Alpes region

## Abstract

Bovine besnoitiosis is a parasitic disease caused by the parasite *Besnoitia besnoiti*. It was classified as an emerging disease by EFSA in 2010, due to the appearance of new cases in several European countries. The clinical presentation can be acute or chronic, but most animals remain asymptomatic, acting as reservoirs. The disease is associated with important economic losses and strict control measures are necessary to mitigate the spread of infection. In recent years, the Health Defense Group of the Auvergne Rhône-Alpes region in France has implemented a voluntary program to monitor the presence of the infection in dairy cattle, through the testing of bulk milk samples collected in dairy farms. The aim of this study was to assess the distribution and evolution of the disease from January 1, 2020 to July 1, 2023. The official results of bulk milk tests from 7,552 dairy farms in all 12 departments of the region were analyzed. The milk samples were tested in certified laboratories using an indirect ELISA (ID Screen^®^ Besnoitia Milk Indirect, IDVET laboratory). Descriptive statistics, sample proportions and disease prevalence were calculated for each year and department. The effects of department, year of analysis, farm type, and number of analyses on the risk of a farm testing positive was investigated. In all the departments, more than 96.2% of active farms were tested in at least 1 year of the study period, with most departments conducting testing annually. Of the farms tested, 12% were positive in at least 1 year. Prevalence varied significantly over the study period and between departments (*p* < 0.01). The lowest prevalence was observed in the Rhône department in 2021, at 0.36%, while the highest prevalence of 23.44% was recorded in the Savoie department in the same year, based on a testing coverage of 97.5% and 100% of active farms, respectively. The probability of a farm testing positive was 4.1% when only one sample was collected annually, increasing to 7.3%, 12.6% and 20.9%, when two, three and four samples were collected respectively. Farms with mixed production types had a higher probability of testing positive. The present study reinforces the importance of regular, repeated testing and focused monitoring of farms with higher risk profiles, such as mixed-production operations, to effectively control and manage bovine besnoitiosis.

## 1 Introduction

Bovine besnoitiosis is a parasitic disease caused by *Besnoitia besnoiti*, a protozoan belonging to the phylum Apicomplexa, family Sarcocystidae and subfamily Toxoplasmatinae (42). The disease was first documented in 1884 by Cadéac in Southern France, where it was initially called “Éléphantiasis *du boeuf”* (Elephant skin disease of cattle) due to the characteristic thickening and wrinkling of the skin (1). In 1912, in the Pyrenees, Besnoit and Robin discovered that this disease was caused by a parasite, and described it as Sarcosporidiosis, despite some differences relative to the disease caused by *Sarcocystis* spp. (41). The emergence of bovine besnoitiosis in Europe, in Portugal and France, at the turn of the 20th century, may be linked to the importation of animals from Africa (2).

The mode of transmission of bovine besnoitiosis under natural conditions is still unclear. The existence of a heteroxenous cycle with a yet unidentified definitive host is generally assumed (3). In a typical apicomplexan prey-predator life cycle, after preying on the infected intermediate host (IH) the definitive host sheds oocysts into the environment. These sporulate and infect the IH by fecal-oral route. In the IH, the sporozoites differentiates into tachyzoites, which replicate in the endothelial cells of blood vessels and, sometimes, in monocytes and neutrophils. At a later stage of infection, tachyzoites differentiate into bradyzoites, encysting in cells such as fibroblasts, myofibroblasts, endothelial cells and smooth muscle cells (4). A recent study detected the presence of *Besnoitia* spp. DNA in fecal samples from red foxes, suggesting a potential role of these carnivores in the epidemiology of *B. besnoiti*, but the results require further investigation (5). Wild ruminants may act as intermediate hosts (IH), however, the low prevalences found indicate that these animals play a minor role in the transmission of the parasite to cattle (6).

Transmission through hematophagous insects has been implicated as the most likely mechanism and was demonstrated experimentally for Diptera of the genera *Glossina, Tabanus* and *Stomoxys* (7). However, other transmission hypotheses, such as direct contact with ruptured cysts on mucous membranes or open skin lesions of infected animals cannot be ruled out (8). Iatrogenic transmission resulting from the reuse of hypodermic needles between animals also poses a risk of indirect transmission (7).

The multiplication of tachyzoites and the encystation of bradyzoites correspond, respectively, to the acute and chronic phases of the disease (8).

Ten different species of *Besnoitia* were identified (9) and divided into two phylogenetically different groups: one that includes the species *B. akodoni, B. darlingi, B. oryctofelisi* and another that includes the species *B. besnoiti, B. bennetti, B. tarandi* and B. jellisoni (10). Four species infect rodents (*B. akadoni, B. neotomofelis, B. jellisoni, B. wallacei*), one infects lagomorphs (*B. oryctofelisi*), and one infects marsupials and lizards (*B. darlingui*), while the remaining four (*B. besnoiti, B. tarandi, B. caprae* and *B. bennetti*) infect domestic and wild ungulates, and respectively cattle and antelopes, reindeer and musk ox, goats, and donkeys and horses (11). *B. besnoiti* also affects wild species such as blue wildebeest, African lion, impala and kudu in Africa (12).

The disease was known to be endemic in several countries of sub-Saharan Africa, Asia and the Middle East (13), but since 2010, it has been recognized as re-emerging in Europe due to an increase in the number of cases and geographical expansion (14). It now extends to Portugal, Spain, Italy, Germany, Switzerland, Croatia, Greece, Hungary, Belgium and Ireland (3, 15–18, 39, 40). Cases have also been reported in Israel, Russia, China, Kazakhstan, South Korea, Uzbekistan and Venezuela (19).

Clinically, bovine besnoitiosis is characterized by a succession of three phases: a febrile phase, with hyperthermia (40.8 to 41.6°C) and non-specific clinical signs that can go unnoticed, such as depression, enlarged lymph nodes, weight-loss, photophobia, nasal and ocular discharge, and skin hyperesthesia (20); an edema or anasarca phase, with general swelling of the whole body, due to an increase in vascular permeability caused by the rapid multiplication of tachyzoites in endothelial cells (38); and a scleroderma phase (chronic phase), characterized by the formation of sand-like cysts containing bradyzoites in the mucous membranes and connective tissues, visible to the naked eye especially on the sclera conjunctiva (pathognomonic sign) and the vulval mucosa (38). Most infected animals remain asymptomatic, with only a small proportion developing clinical signs—ranging from 1–10% in endemic areas to 15–20% in emerging regions (21). A mortality rate of up to 10% has been observed (7). Asymptomatic animals represent a threat for the herd, as they can act as a source of infection for healthy animals (9).

Due to the initial non-specific clinical signs, several differential diagnoses should be considered for bovine Besnoitiosis, including malignant catarrhal fever, bovine granulocytic ehrlichiosis, bluetongue fever, bovine respiratory disease, photosensitization, scabies, and zinc deficiency (2).

Bovine besnoitiosis adversely affects animal welfare and has a medium to high economic impact (11), resulting primarily from loss of body condition, decreased milk production, transitory to permanent infertility in males, abortions and decreased quality of hides (8). Effective control remains challenging. A live attenuated vaccine is available in some countries, but not in Europe. Sulfonamides can be effective in reducing symptoms in the acute phase but are ineffective in the chronic phase of the disease as the parasite is already encysted (21, 22). Treatment is in general discouraged because animals remain subclinically infected and may act as a reservoir for the disease.

The disease can be diagnosed by direct or indirect methods. Direct diagnosis in chronically infected animals involves histopathology of skin biopsies, which is considered the gold standard. Skin samples must be 8 mm in diameter (23). Diagnosis by conventional polymerase chain reaction (PCR) or real-time (rt-PCR) from skin samples, has a higher sensitivity than indirect methods in acutely infected cows (9). Indirect methods are based on the detection of specific antibodies in the serum of animals with clinical or subclinical infection (9). The Immunofluorescent Antibody Test (IFAT) has greater sensitivity and specificity than the Enzyme-Linked Immunosorbent Assay (ELISA) (24). There are no cross-reactions between *B. besnoiti* tachyzoites and anti-*T. gondii* and anti-*N. caninum* antibodies at the commonly used cut-offs (9). The ELISA test is also widely used in epidemiological studies. When a serum tests positive in an ELISA, confirmation by Western-blot test is generally recommended. This increases the detection of true positives, by avoiding possible false-positive results due to cross-reactions with other Apicomplexan parasites (e.g., *Neospora* spp. or *Sarcocystis* spp.) (25). The combination of serological tests followed by PCR also increases the probability of detecting the disease (8).

**Figure 1:**
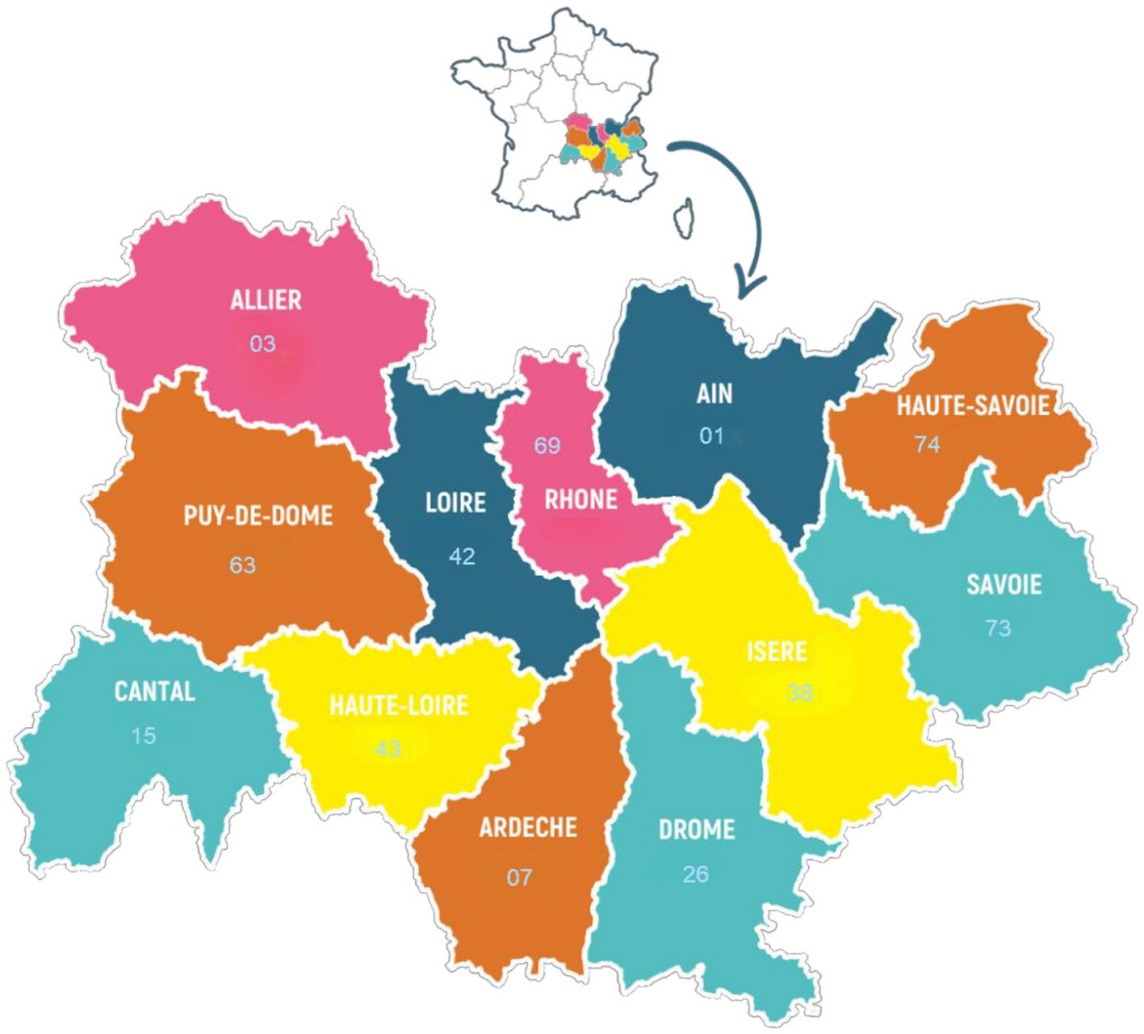
Geographic representation of the Auvergne Rhône-Alpes region, France (https://ffessmaura.fr/uploads/menus/524/kVBTtSAdXGLKheP4iDzumCqU2ORcYbg1M7pv9NnZw38/media/resized30062024114653carteregionaura1051x964.png).

Auvergne Rhône-Alpes, one of France's 18 administrative regions, is geographically diverse and large, extending over almost 70,000 km^2^ and comprises 12 departments (Figure 1). This region is known for its varied landscapes, from the Jura and Alps mountains, to the volcanic Massif Central, the great lakes, the plains of the departments Rhône, Isère, Allier or Loire, and mid-mountain areas. Approximately 70% of the region's surface is classified as mountainous, while about three quarters consist of natural green areas alongside highly developed agricultural activities (26).

In recent years, an increasing number of sick animals and infected cattle farms have been reported in the Auvergne Rhône-Alpes region. This region is not considered to be one of the most affected by bovine besnoitiosis in the country, but it is contiguous with neighboring departments that are known to be endemic. This prompted the region's Health Defense Group (Groupement de Défense Sanitaire, GDS) to implement monitoring procedures on local farms. Since 2021, bovine besnoitiosis screening has been highly recommended—though not mandatory—in several departments (Ain, Ardèche, Drôme, Isère and Rhône), with diagnostic costs fully or partially supported by the GDS. Screening was carried out in parallel with official disease control plans.

Given this context, it has become important to characterize the distribution and evolution of the infection in all departments of the region. This information will help inform the GDS about future needs and strategic decisions regarding bovine besnoitiosis management.

The objective of this study was to characterize the presence of *B. besnoiti* infection in dairy cattle farms in the Auvergne Rhône-Alpes region through a retrospective analysis of screening test results performed as part of the monitoring programs conducted by the GDS. Specifically, the study aimed to determine the prevalence of infected farms, using the departments of the region as geographic units, describe the temporal evolution of the infection from January 1, 2020 to July 1, 2023 and identify potential risk factors associated with the disease.

## 2 Materials and methods

This study received a favorable opinion from the Ethics Committee of the Escola Universitária Vasco da Gama. The data used are the property of the Health Defense Group of the Auvergne Rhônes-Alpes region and were provided for exclusive use in the present study. Data can be made available upon request.

### 2.1 Selection of farms and data collection

This retrospective study involved the analysis of bulk milk test results for *B. besnoiti* from dairy farms surveyed across the 12 departments of the Auvergne Rhône-Alpes Region. Samples were obtained during the period from January 1, 2020 to July 1, 2023. In each department, official disease control and prophylactic programs are conducted annually in all farms between the beginning of October and the end of April. These plans target Infectious Bovine Rhinotracheitis, Brucellosis, Leucosis, Tuberculosis and Hypodermosis. During interventions, one bulk milk sample was collected in each farm, at the time of milk collection and transport to the industry. Testing was performed within the scope of the official programs. The number of milk samples obtained in each farm during the study period varied depending on the official control schedule for other diseases, monitoring visits and the health status of each farm. Samples were tested in the officially designated laboratory for both the mandatory diseases and bovine besnoitiosis. Farms were tested for *Besnoitia* either as part of systematic screening, through initiatives of the GDS, with producer consent, or upon specific farmer request. In the first case, the costs were supported by the GDS of each department. All dairy farms in each Department were eligible for testing, with varying numbers of farms tested annually throughout the study period. All lactating animals producing milk (deemed fit for human consumption) at the time of the official visit were included in the composite bulk milk sample. The sample was collected after at least one complete (12 h) milking. These animals were, in general, older than 24 months. Individual animal breed information was not collected; however, based on official GDS records, the Montbéliard breed predominated, followed by the Prim'Holstein breed. Male animals were excluded from the study.

The samples were sent to the laboratory immediately after collection, frozen and tested within a month (Agrolabs laboratory, for 9 of the 12 departments in the region; LDA laboratory, in the Ain department; LIDAL laboratory, in the Savoie and Haute-Savoie departments). All laboratories used the same procedures and tests. The results were communicated monthly and recorded by the official GDS services in their own database (database for non-regulatory diseases, AGDS). Serological testing was performed using the ID Screen^®^ *Besnoitia* Milk Indirect ELISA (IDVET Laboratory, 310 Rue Louis Pasteur 34,790 Grabels, France) according to the manufacturer's instructions.

The ELISA results between July 1, 2023 and January 1, 2020 in the AGDS database were retrospectively extracted to Excel (Microsoft Excel, version 2016). All dairy cattle farms (farms with adult dairy cows) or mixed farms (farms with both beef and dairy cows) registered in each department of the Auvergne Rhône-Alpes Region, were included in the study.

The data collected included the identification of active farms in each year, the geographic location of farms by department, the type of production (milk or mixed), the number of samples tested per farm and the number of samples with positive results in each year. The number of adult animals present in each farm at the date of data extraction (July 2023), was also recorded.

### 2.2 Statistical analysis

For statistical analysis, the SAS^®^ 9.4 program was used (27). Initially, the descriptive statistics of the available data were determined using PROC FREQ from the SAS program. The frequencies of farms with positive results were determined by department, according to the type of production and by year (2020, 2021, 2022, and 2023). The average percentage of positive farms per department and per year, the respective standard errors and confidence intervals, were calculated using PROC MEANS from the SAS program. The analysis of variance of the prevalence of bovine besnoitiosis was carried out using PROC GLM of the SAS program, with a model that included the effects of the department and the year of sample collection and, subsequently, the least square means of the prevalence per department and per year and the respective *t*-tests for the differences of the LS-means. Prevalence was calculated using the proportion of positive farms in relation to the total number of farms tested in the period considered. Thus, for each year and in each department, the prevalence of the disease and its respective 95% confidence interval were calculated. The proportion of farms tested per department and per year was calculated, dividing the number of farms tested by the total number of active farms in each department, in the respective year.

Maps were created with the Microsoft Excel^®^ program to illustrate the distribution of farms by department and the respective proportions of farms tested, for each year. The number and proportion (%) of new positive farms, in relation to the previous year, was determined. The number and proportion of farms with at least one sample with a positive result in the total study period (4 years); the number and proportion of farms that were no longer positive, in relation to the previous year; the number and corresponding proportion of farms that were always negative in the total period of the study, and the number and percentage of farms at risk were also calculated.

The results of a total of 35,334 ELISA tests carried out during the study period (2020 to 2023), were considered (data collected until July 1, 2023).

The probability of farms being positive for bovine besnoitiosis was investigated through logistic regression analysis and the Wald test, with PROC LOGISTIC from the SAS program, considering the farm's positivity as a dependent variable and, as independent explanatory variables, the department, the number of results available, the number of animals on the farm and the year of observation. The comparative investigation of the risk of a farm being positive for bovine besnoitiosis was carried out by calculating probability ratios (Odds Ratios), considering several risk factors, namely, the department where the farm is located, the year of analysis and the type of farm (dairy farm vs. mixed farm), the total number of analyzes carried out in each farm (only for the year 2022) and the total number of animals on the farm (only for the year 2023).

## 3 Results

**Figure 2:**
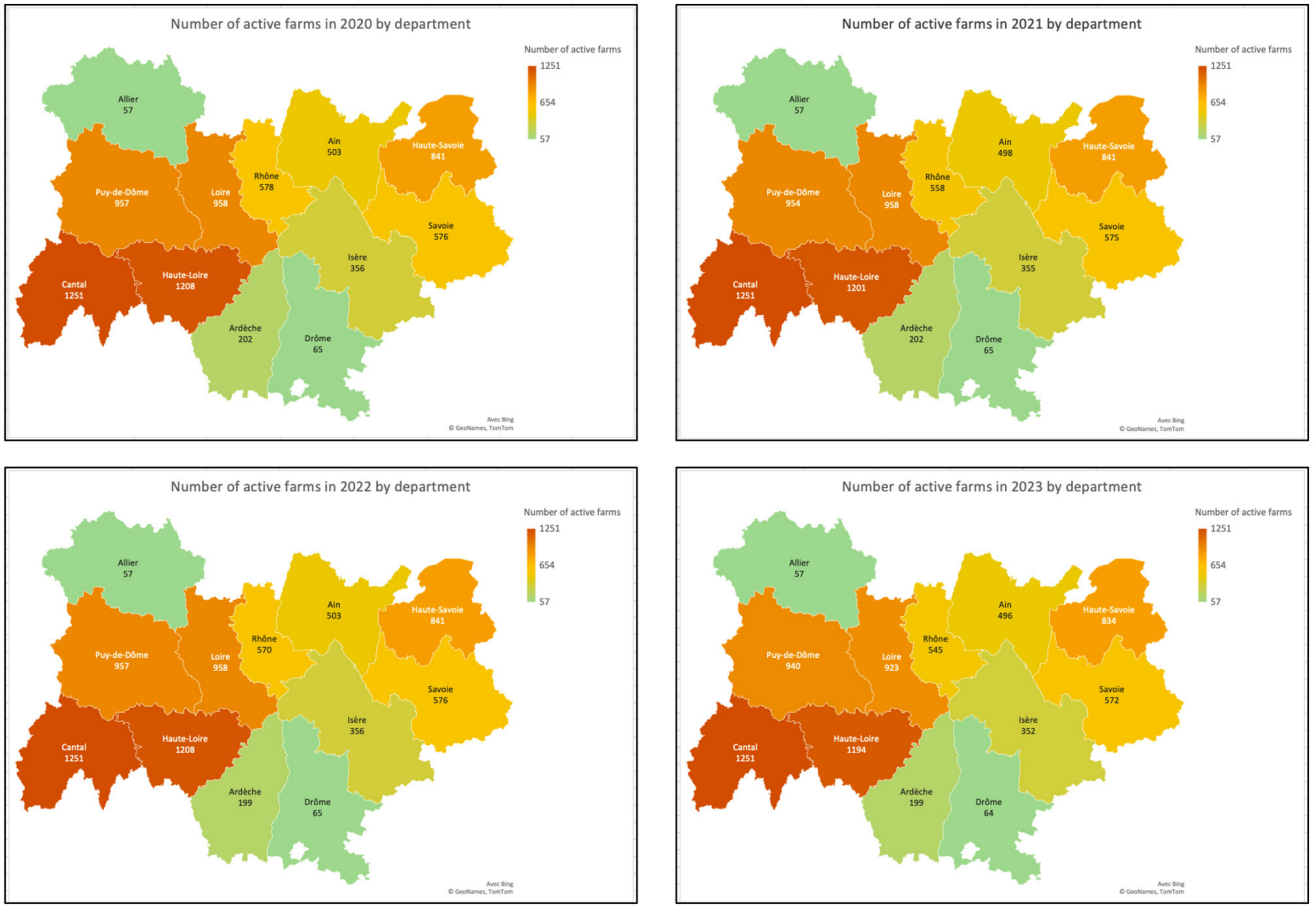
Geographic representation of the distribution of the number of active farms in 2020, 2021, 2022, and 2023, by department.

During the study period, some farms ceased their activities and others started or converted to beef cattle production. Additionally, the proportion of farms monitored by department in relation to the total number of farms varied each year, depending on the priorities set out in the official plans. ELISA results were retrieved for a total of 7552 dairy and mixed farms in the region. The number of adult cows in these farms in 2023 was 800,391. These farms correspond to the total number of dairy/mixed cow farms registered in the 12 departments in 2020. The number of active farms by geographic location in the department and by year is shown in Figure 2 and detailed in Table 1. Sampling and testing of farms, by department and year (Table 2) varied along the study period. A testing coverage of 48.2%, 90.0%, 91.6%, and 55.2% of all existing farms in the Auvergne-Rhône-Alpes region was achieved in 2020, 2021, 2022, and 2023, respectively (Table 1).

**Table 1:** Number of active farms and proportion of farms tested by department and per year.

**Department**	**Number of active farms (A) and proportion (%) of farms tested (B)**							
	**2020**		**2021**		**2022**		**2023**	
	**(A)**	**(B)**	**(A)**	**(B)**	**(A)**	**(B)**	**(A)**	**(B)**
Ain	503	92.80%	503	98.20%	498	98.40%	496	98.20%
Allier	57	0	57	0	57	100%	57	0
Ardèche	202	95%	202	97%	199	99.50%	199	95.50%
Cantal	1,251	7.50%	1,251	96.20%	1,251	93.30%	1,251	85.90%
Drôme	65	0	65	98.50%	65	98.50%	64	0
Isère	356	95.20%	356	97.50%	355	98.90%	352	1.70%
Loire	958	99.40%	958	99.90%	958	100%	923	99.20%
Haute-Loire	1,208	90.10%	1,208	99.30%	1,201	95.90%	1,194	93.80%
Puy-de-Dôme	957	0	957	96.30%	954	20.10%	940	87.60%
Rhone	578	98.10%	570	97.50%	558	98.20%	545	97.10%
Savoie	576	0	576	100%	575	97.60%	572	1.70%
Haute-Savoie	841	0	841	99.90%	841	98.30%	834	2%
TOTAL	7,552	48.20%	7,544	90.00%	7,512	91.60%	7,427	55.2%

**Table 2:** Number of farms sampled per department and per year.

**Department**	**2020**	**2021**	**2022**	**2023**
Ain	467	494	490	487
Allier	0	0	57	0
Ardèche	192	196	198	190
Cantal	94	1,203	1,167	1,074
Drôme	0	64	64	0
Isère	339	347	351	6
Loire	952	957	958	916
Haute-Loire	1,088	1,199	1,152	1,120
Puy-de-Dôme	0	922	192	823
Rhone	567	556	548	529
Savoie	0	576	561	10
Haute-Savoie	0	840	827	17
TOTAL	3,699	7,354	6,565	5,172

**Figure 3:**
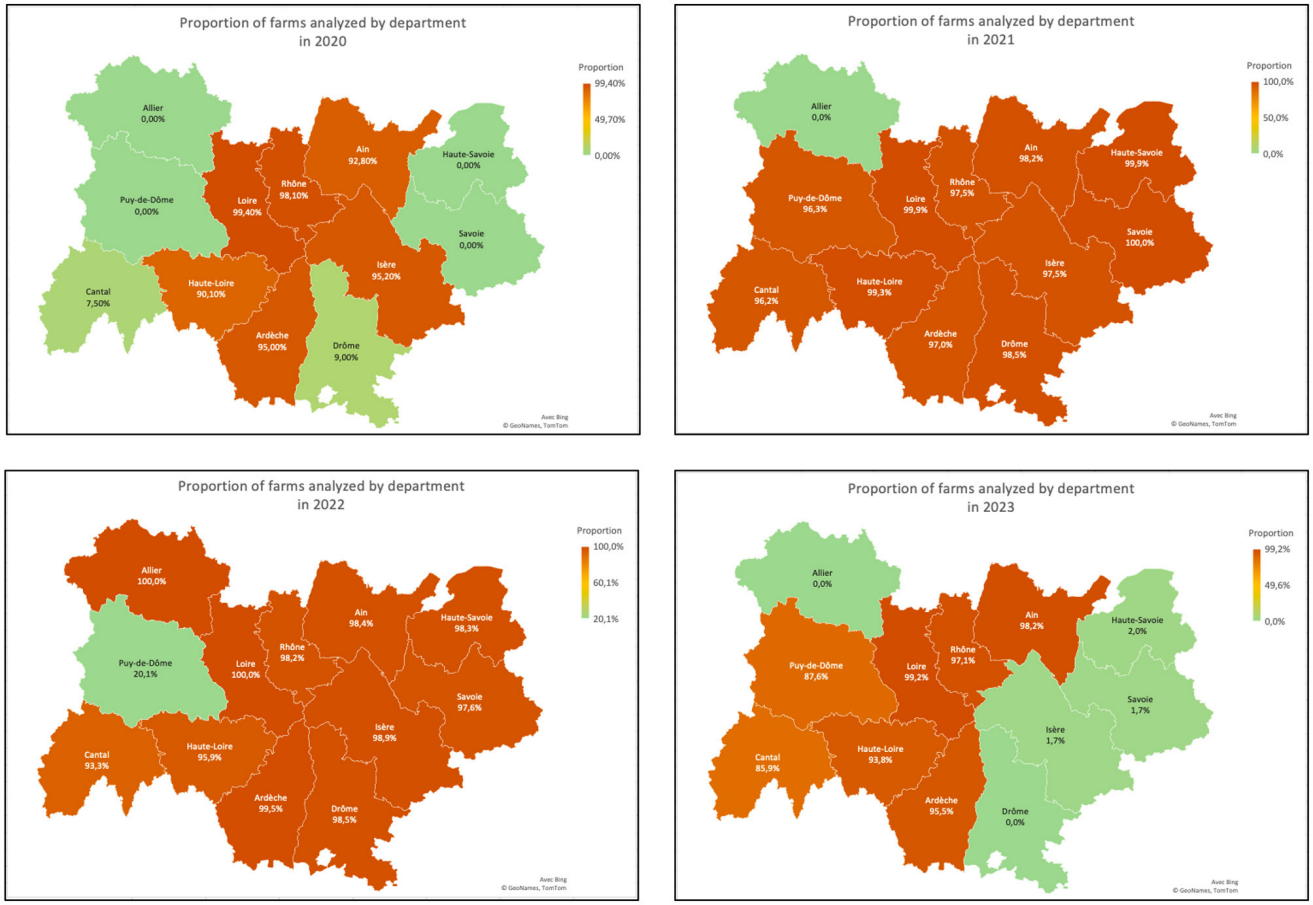
Proportion of farms sampled in 2020, 2021, 2022, and 2023, by department.

**Figure 4:**
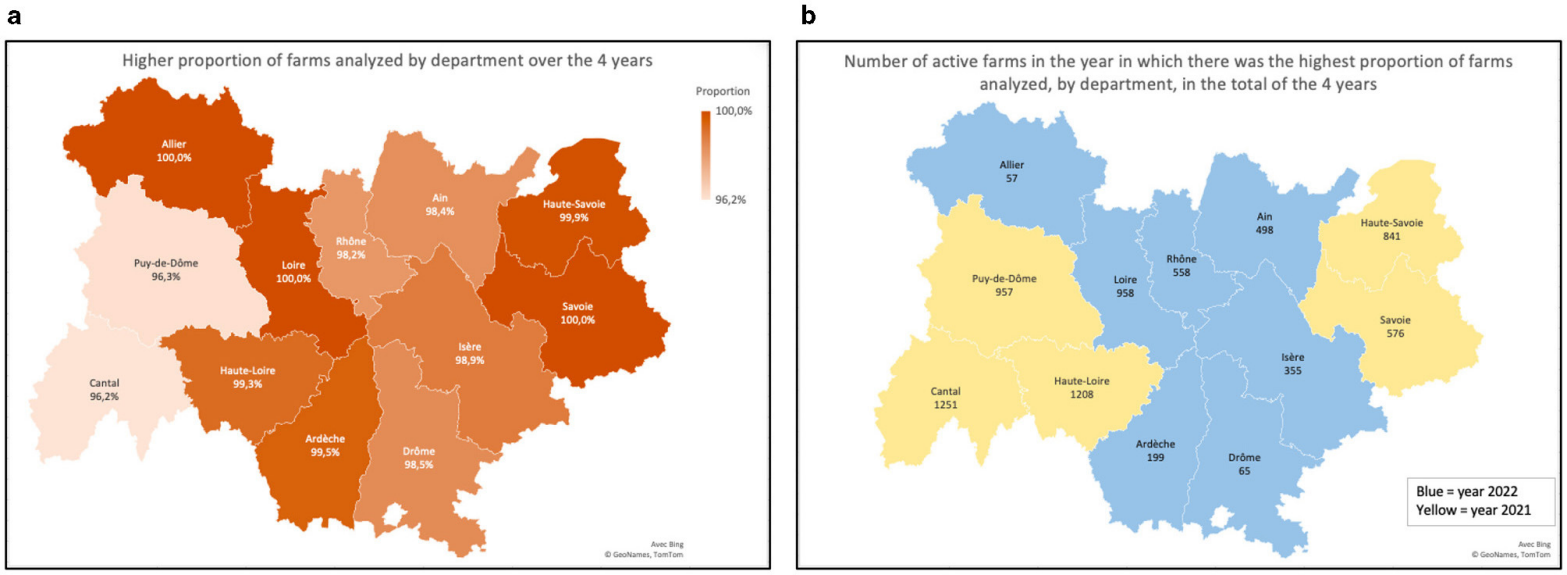
**(a)** Higher proportion of farms tested annually in each department, observed during the total period of the 4 years of the study; **(b)** Number of active farms registered in the GDS and correspondent year of the highest proportion of farms tested in each department.

The proportion of sampled farms is characterized by department and year in Figure 3. Figure 4 depicts the highest proportion of farms tested in each department, during the 4-year follow-up, and the corresponding number of active farms in each department.

Of the farms observed, 6,230 were dairy and 1,281 were mixed. Additionally, 39 of the dairy farms changed their production type to suckler production, and two to fattening, in the last year of study.

**Figure 5:**
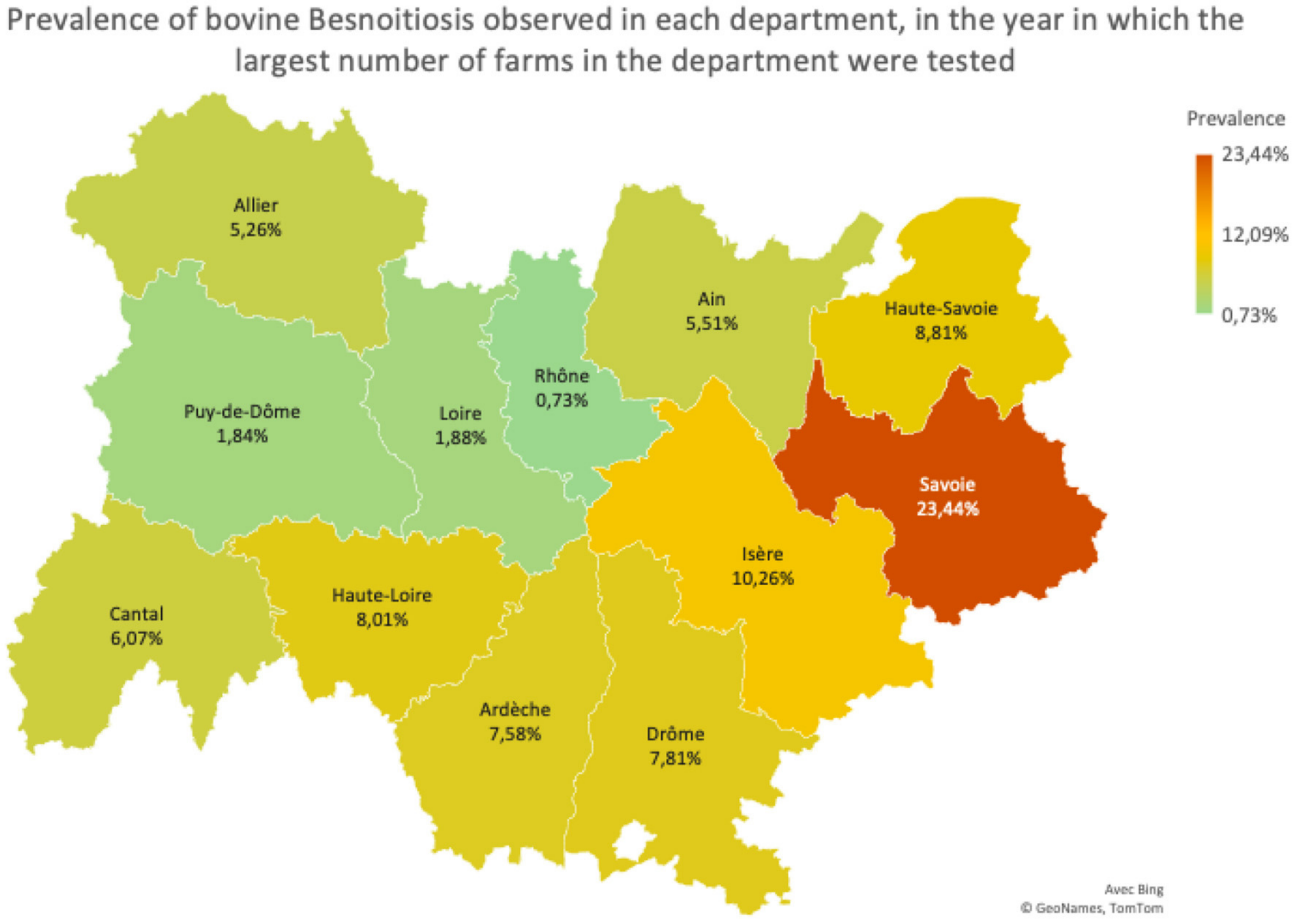
Prevalence observed in each department, in the year in which the largest proportion of active farms were tested.

Samples were taken from bulk tanks, which hold milk from all lactating cows on the farm. During the study period, a total of 35,334 milk samples were tested. Out of the total samples tested, 33,301 (94.25%) were negative, while 2,033 (5.75%) tested positive. A significant effect (*p* < 0.01) of the department on the prevalence of *B. besnoiti* infection by year is shown in Tables 3, 4. Ardèche was the department with the highest prevalence in the year 2020 (10.94%). In 2021 and 2022 the prevalence was highest in Savoie, with 23.44 % and 17.83% of infected farms, respectively. In 2023, until the date of data extraction, the department of Isère had the highest prevalence (16.67%). The map in Figure 5 illustrates the distribution of prevalence by department, in the year in which the highest proportion of farms was tested (Figure 5).

**Table 3:** Prevalence of bovine besnoitiosis by department and by year (95% CI, 95% confidence interval; *N*° obs (%): number of observations and proportion relative to active farms).

**Department**	**Year**	**Prevalence**			
		**Average**	**95% CI**		*N*°**obs (%)**
Ain	2020	1.50%	−0.15%	3.15%	467 (92.8%)
	2021	2.23%	0.62%	3.83%	494 (98.20%)
	2022	5.51%	3.90%	7.12%	490 (98.40%)
	2023	4.52%	2.90%	6.14%	487 (98.20%)
Allier	2020	-	-	-	0 (0%)
	2021	-	-	-	0 (0%)
	2022	5.26%	2.98%	8.32%	57 (100%)
	2023	-	-	-	0 (0%)
Ardèche	2020	10.94%	7.07%	14.81%	192 (95%)
	2021	8.67%	4.84%	12.50%	196 (97%)
	2022	7.58%	3.77%	11.39%	198 (99.5%)
	2023	5.26%	1.37%	9.15%	190 (95.5%)
Cantal	2020	7.45%	1.99%	12.91%	94 (7.5%)
	2021	6.07%	4.54%	7.59%	1,203 (96.2%)
	2022	7.46%	5.91%	9.00%	1,167 (93.3%)
	2023	10.61%	9.00%	12.23%	1,074 (85.9%)
Drôme	2020	-	-	-	0 (0%)
	2021	7.81%	1.44%	14.18%	64 (98.5%)
	2022	6.25%	−0.12%	12.62%	64 (98.5%)
	2023	-	-	-	0 (0%)
Isère	2020	8.55%	5.58%	11.53%	339 (95.2%)
	2021	6.63%	3.68%	9.57%	347 (97.5%)
	2022	10.26%	7.33%	13.18%	351 (98.9%)
	2023	16.67%	−5.72%	39.05%	6 (1.7%)
Loire	2020	2.63%	1.72%	3.53%	952 (99.4%)
	2021	2.09%	1.19%	2.99%	957 (99.9%)
	2022	1.88%	0.98%	2.78%	958 (100%)
	2023	1.64%	0.72%	2.56%	916 (99.2%)
Haute-Loire	2020	6.71%	5.10%	8.32%	1,088 (90.1%)
	2021	8.01%	6.47%	9.54%	1,199 (99.3%)
	2022	10.94%	9.37%	12.50%	1,152 (95.9%)
	2023	6.34%	4.75%	7.93%	1,120 (93.8%)
Puy-de-Dôme	2020	-	-	-	0 (0%)
	2021	1.84%	0.75%	2.94%	922 (96.3%)
	2022	6.25%	3.84%	8.66%	192 (20.1%)
	2023	3.52%	2.36%	4.69%	823 (87.6%)
Rhone	2020	1.06%	0.30%	1.82%	567 (98.1%)
	2021	0.36%	−0.41%	1.13%	556 (97.5%)
	2022	0.73%	−0.05%	1.51%	548 (98.2%)
	2023	1.32%	0.53%	2.11%	529 (97.1%)
Savoie	2020	-	-	-	0 (0%)
	2021	23.44%	20.14%	26.74%	576 (100%)
	2022	17.83%	14.48%	21.17%	561 (97.6%)
	2023	10.00%	−15.05%	35.05%	10 (1.7%)
Haute-Savoie	2020	-	-	-	0 (0%)
	2021	8.81%	6.93%	10.69%	840 (99.9%)
	2022	8.32%	6.29%	10.35%	721 (98.3%)
	2023	6.56%	1.62%	11.50%	122 (2%)

**Table 4:** Proportion of positive new farms, compared to the previous year.

**Department**	**2021 vs. 2020 %(n)**	**2022 vs. 2021 %(n)**	**2023 vs. 2022 %(n)**
Ain	1.9% (9)	4.5% (22)	1.6% (8)
Allier	0.0% (0)	– (3)	– (0)
Ardèche	3.6% (7)	3.6% (7)	1.5% (3)
Cantal	72.3% (68)	4.2% (51)	5.9% (69)
Drôme	(5)	1.6% (1)	0% (0)
Isère	0.9% (3)	4.6% (16)	0% (0)
Loire	1.5% (14)	1.4% (13)	1.3% (12)
Haute-Loire	3.6% (39)	5.4% (65)	1.1% (13)
Puy-de-Dôme	(17)	0.9% (8)	9.9% (19)
Rhone	0.4% (2)	0.7% (4)	0.9% (5)
Savoie	(135)	5.4% (31)	0.2% (1)
Haute-Savoie	(74)	4.0% (34)	0.0% (0)
TOTAL	6.4%	7.6%	5.2%

The number of farms that tested positive for the first time, compared to the previous year was determined (Table 5). In the year 2021 there was an increase of 10.1% of farms that became positive. In 2022, an additional 3.5 % of newly positive farms compared to the previous year was observed. Up to the end of the study on July 1, 2023 there were already 2% more positive farms.

**Table 5:** Absolute and relative frequencies of farms that were positive at least once and of farms that were always negative, over all 4 years.

**Department**	**Farms that tested positive at least 1 year (%)**	**Farms that remained negative in the 4 years (%)**
Ain	8.9% (45)	91.1% (458)
Allier	5.3% (3)	94.7% (54)
Ardèche	17.3% (35)	82.7% (167)
Cantal	14.9% (186)	85.1% (1,065)
Drôme	9.2% (6)	90.8% (59)
Isère	13.2% (47)	86.8% (309)
Loire	6.5% (62)	93.5% (896)
Haute-Loire	15.5% (187)	84.5% (1, 021)
Puy-de-Dôme	4.5% (43)	95.5% (914)
Rhone	2.9% (17)	97.1% (561)
Savoie	28.8% (166)	71.2% (410)
Haute-Savoie	12.8% (108)	87.2% (733)
TOTAL	12.0% (905)	88% (6,647)

Significant differences (*p* < 0.01, Table 6) in both the proportion of farms that tested positive in at least 1 year and the proportion of farms that remained negative during the 4 years were observed. The department of Savoie had the highest proportion of positive farms and the corresponding lowest proportion of negative farms during the period. In total, over the period of 4 years, 905 out of 7,552 (12%) farms tested positive at least once, while 6,647 (88%) were always negative (Table 7).

**Table 6:** Absolute and relative frequencies of farms that were no longer positive, in relation to the previous year, in each department.

**Department**	**2020**	**2021**	**2022**	**2023**
Ain		1.1% (5)	1.2% (6)	2.7% (13)
Allier				5.3% (3)
Ardèche		5.7% (11)	4.6% (9)	3.5% (7)
Cantal		2.1% (2)	3.1% (37)	3.6% (42)
Drôme			3.1% (2)	6.3% (4)
Isère		2.7% (9)	0.9% (3)	10.0% (35)
Loire		2.0% (19)	1.6% (15)	1.6% (15)
Haute-Loire		1.5% (16)	2.9% (35)	5.9% (68)
Puy-de-Dôme			1.4% (13)	1.0% (2)
Rhone		1.1% (6)	0.4% (2)	0.4% (2)
Savoie			11.5% (66)	17.8% (100)
Haute-Savoie			4.8% (40)	8.2% (68)
TOTAL		1.8% (68)	3.1% (228)	5.5% (359)

**Table 7:** Absolute and relative frequencies of farms at risk (tested, with negative results).

**Department**	**2020**	**2021**	**2022**	**2023**
Ain	98.5% (460)	97.8% (483)	94.5% (463)	95.5% (465)
Allier			94.7% (54)	
Ardèche	89.1% (171)	91.3% (179)	92.4% (183)	94.2% (179)
Cantal	92.6% (87)	93.9% (1,130)	92.5% (1,080)	89.4% (960)
Drôme		92.2% (59)	93.8% (60)	
Isère	91.4% (310)	93.4% (324)	89.7% (315)	83.3% (5)
Loire	97.4% (927)	97.9% (937)	98.1% (940)	98.4% (901)
Haute-Loire	93.3% (1, 015)	92.0% (1,103)	89.1% (1, 026)	93.7% (1,049)
Puy-de-Dôme		98.2% (905)	93.8% (180)	96.5% (794)
Rhone	98.9% (561)	99.6% (554)	99.3% (544)	98.7% (522)
Savoie		76.6% (441)	82.2% (641)	90.0% (9)
Haute-Savoie		91.2% (766)	91.8% (759)	100.0% (17)
TOTAL	95% (3,531)	94% (6,881)	92% (6,065)	95% (4,901)

Furthermore, the number of farms that became negative in each department (in comparison to the previous year) was calculated. In 2021, 68 became negative. In the years 2022 and 2023, 228, and 359 farms that were previously positive tested negative, respectively (Table 8).

**Table 8:** Absolute and relative frequencies of farms that were no longer positive, in relation to the previous year, in each department.

**Department**	**2020**	**2021**	**2022**	**2023**
Ain		1.1% (5)	1.2% (6)	2.7% (13)
Allier				5.3% (3)
Ardèche		5.7% (11)	4.6% (9)	3.5% (7)
Cantal		2.1% (2)	3.1% (37)	3.6% (42)
Drôme			3.1% (2)	6.3% (4)
Isère		2.7% (9)	0.9% (3)	10.0% (35)
Loire		2.0% (19)	1.6% (15)	1.6% (15)
Haute-Loire		1.5% (16)	2.9% (35)	5.9% (68)
Puy-de-Dôme			1.4% (13)	1.0% (2)
Rhone		1.1% (6)	0.4% (2)	0.4% (2)
Savoie			11.5% (66)	17.8% (100)
Haute-Savoie			4.8% (40)	8.2% (68)
TOTAL		1.8% (68)	3.1% (228)	5.5% (359)

The proportion of farms at risk in each of the departments, given by the proportion of negative farms, was calculated for each year of the study (Table 9). In 2020, there were 95% farms at risk across all departments. In 2021, this percentage dropped to 94%, and to 92% in 2022. Until July 1, 2023, the proportion of farms at risk was 95%.

**Table 9:** Absolute and relative frequencies of farms at risk (tested, with negative results).

**Department**	**2020**	**2021**	**2022**	**2023**
Ain	98.5% (460)	97.8% (483)	94.5% (463)	95.5% (465)
Allier			94.7% (54)	
Ardèche	89.1% (171)	91.3% (179)	92.4% (183)	94.2% (179)
Cantal	92.6% (87)	93.9% (1,130)	92.5% (1„080)	89.4% (960)
Drôme		92.2% (59)	93.8% (60)	
Isère	91.4% (310)	93.4% (324)	89.7% (315)	83.3% (5)
Loire	97.4% (927)	97.9% (937)	98.1% (940)	98.4% (901)
Haute-Loire	93.3% (1,015)	92.0% (1,103)	89.1% (1,026)	93.7% (1,049)
Puy-de-Dôme		98.2% (905)	93.8% (180)	96.5% (794)
Rhone	98.9% (561)	99.6% (554)	99.3% (544)	98.7% (522)
Savoie		76.6% (441)	82.2% (641)	90.0% (9)
Haute-Savoie		91.2% (766)	91.8% (759)	100.0% (17)
TOTAL	95% (3,531)	94% (6,881)	92% (6,065)	95% (4,901)

**Figure 6:**
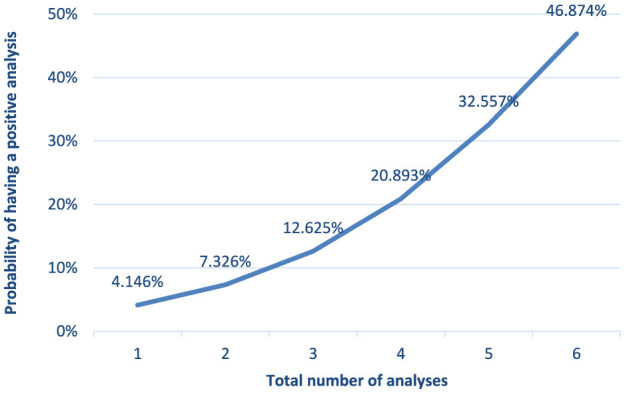
Probability of a farm to have a positive result according to the number of samples available annually (*p* < 0.01).

The likelihood of a farm testing positive for bovine besnoitiosis was significantly associated to the explanatory variables department, study year, number of animals on the farm, number of tests performed and production system. Thus, seropositivity of farms depended significantly of the effect of the department and the number of animals on the farm (*p* < 0.05). With each additional animal on the farm, the probability of a farm being positive increased by 0.002%. However, when considering that the number of animals in 2022 was equal to the number of animals in 2023, there was no significant association between the number of animals on the farm and the probability of having a positive result in 2022. Significant differences between years were observed in some departments (Table 4). In the departments of Ain, Cantal and Pui-de-Dome, prevalences increased significantly during the study period. In Haute-Loire, the prevalence rose significantly (*p* < 0.05) from 6.7% in 2021 to 10.9% in 2022 and decreased to 6.34% in the first semester of 2023. In the department of Savoie the prevalence significantly declined (*p* < 0.05) from 23.4% in 2021 to 10% in 2023. In all departments, the probability of a farm having a positive outcome depended significantly (*p* < 0.01) on the number of samples collected. On average, for each additional sample result available for a given farm, the probability of being positive increased by 82.8%. The probability of a farm having a positive result was 4.1% when only one sample was available, increasing to 7.3%, 12.6%, and 20.9%, respectively, when two, three and four samples were collected annually (Figure 6). The probability of a farm being positive was higher for the mixed type of production in each year, except for 2020, as well as for the global study period. During the study period, the odds ratios (OR) of test positivity for mixed farms was 1.47 times higher than for dairy farms (Table 10). The OR of test positivity were calculated for each department (Table 10), using the Rhône Department as the reference as it showed the lowest probability of positive farms. The risk of having positive farms was 20.4 times higher in the Department of Puys-de-Dome compared to the Department of Rhône. The OR of a farm testing positive was also calculated for each year, considering 2021 as the reference year (the year with the highest number of tests carried out). The OR 2020 vs. 2021 was 1.1, the OR 2022 vs. 2021 was 1.26 and the OR 2023 vs. 2021 was 1.28. Farms exhibited the lowest risk of testing positive in 2021 and the highest risk in 2023 (Table 10).

**Table 10:** Odds Ratio estimates of the risk of a farm being positive for bovine besnoitiosis by type of farm, year, department, total number of analyzes carried out in each farm (only for the year 2022) and total number of animals on the farm (only for the year 2023).

**Effect**		**OR estimate**	**95% Wald confidence limits**		**Wald Chi-Sq value**
**Type of farm**	**Mixed vs. dairy farm**	**1.471**	**1.291**	**1.676**	**22.426** ^**^
Year (each year vs. 2021)	2020 vs. 2021	1.105	0.928	1.317	0.531^ns^
	2022 vs. 2021	1.260	1.132	1.402	4.754^*^
	2023 vs. 2021	1.287	1.120	1.480	4.478^*^
Department (each department vs. Rhone)	Ain vs. Rhone	5.885	1.699	20.382	0.583 ^ns^
	Allier vs. Rhone	8.072	4.979	13.089	0.014 ^ns^
	Ardèche vs. Rhone	9.052	5.816	14.09	10.170^**^
	Cantal vs. Rhone	6.321	3.407	11.724	42.172^**^
	Drôme vs. Rhone	8.043	5.063	12.777	0.402 ^ns^
	Isère vs. Rhone	1.659	1.035	2.659	13.899^**^
	Loire vs. Rhone	8.297	5.346	12.878	119.305^**^
	Haute-Loire vs. Rhone	3.575	2.18	5.865	30.629^**^
	Puy-de-Dôme vs. Rhone	20.494	13.055	32.172	11.144^**^
	Savoie vs. Rhone	7.144	4.517	11.299	240.635^**^
	Haute-Savoie vs. Rhone	5.885	1.699	20.382	7.730^**^
Total n° of analysis in each farm		1.828	1.449	2.306	25.854^**^
Total n° of animals on the farm		1.002	1	1.003	3.854^*^

^ns^Non significant (*p* < 0.05); ^*^Significant at *p* < 0.05; ^**^Significant at *p* < 0.01.

## 4 Discussion

The aim of this study was to characterize the prevalence of bovine Besnoitiosis on dairy farms in the Auvergne Rhone Alps region between January 1, 2020 and July 1, 2023. Overall, the results indicate that prevalence fluctuates over time, with higher rates observed in Ardèche (2020), Savoie (2021–2022), and Isère (2023). For several years, this illness was ignored, but with the increase in the number of cases in the last two decades, studies on the prevalence of the disease and its spread are essential to try to limit its expansion.

The prevalence of bovine besnoitiosis is associated to some risk factors, such as the age of animals and breed (6). While transmission via the sexual route has been hypothesized, parasite DNA has not been detected in semen from chronically infected bulls (43). Coelho et al. (6) reported a higher seroprevalence in Salers and Charolais breeds compared to crossbred cows, suggesting differences in susceptibility to infection. Beef cattle are in general the most affected, although, in some instances, the number of cases may be higher in dairy cows (28). The increased susceptibility of beef cattle breeds may be linked to the extensive mode of production, as these animals graze on pastures for most of the year. Outdoor reared cattle, for instance, may face a greater risk of infection with *Neospora caninum*, a phylogenetically related parasite, due to a heightened exposure to oocysts contaminating the environment (29). In the case of *B. besnoiti* however, although a heteroxenous life cycle is in general postulated due to the parasite's coccidial nature, experimental infections of putative definitive hosts have not yet resulted in oocyst shedding, and molecular evidence of the parasite in carnivore feces (5), requires further confirmation. Conversely, prolonged exposure to hematophagous insects on pastures, especially during the warmer spring and summer months, when insects are more active, has been associated with an increased prevalence of *B. besnoiti* infection. With no known biological vector, mechanical transmission has been demonstrated experimentally for biting flies, such as *Stomoxys calcitrans, Glossina brevipalpis* and tabanids (7). Despite the short-range nature of transmission—due to *B. besnoiti* surviving only briefly on the vector's mouthparts—outdoor breeding increases proximity to potentially infected animals from neighboring farms or wild ruminants, which may serve as reservoirs or intermediate hosts (25). In addition to mechanical transmission by hematophagous vectors, iatrogenic transmission has been suggested as another possible route for the spread of *B. besnoiti*, e.g., through the reuse of contaminated needles (7). Age also seems to be relevant, as older cows have higher seropositivity (95% seropositivity in cows with more than four lactations, compared to 61% seropositivity in first-lactation cows) (17). Furthermore, cows aged between 1 and 3 years, in addition to those over 7 years of age, with prolonged and continuous exposure to the vector, are at most risk (6). The disease rarely affects animals under 6 months of age, although calves can be seropositive after ingesting colostrum from positive mothers (30). Diezma-Díaz et al. (31) reported a case of besnoitiosis in a calf < 6 months old.

Topographic factors also seem to influence the prevalence of bovine besnoitiosis, with animals pastured at altitudes above 600 m showing a higher seroprevalence (38).

During the study period, significant differences in prevalence between departments of the same region were observed. In fact, the disease is progressing in France, following a gradient from South to North (32). Some departments implemented different sanitation measures to control disease geographic expansion, as is the case of Ardèche, Loire and Rhône, where the annual prevalence decreased during the study period. Such measures included: annual or biannual testing of bulk tank milk, followed by recommendations to screen the entire herd in order to cull seropositive animals in positive farms, financial compensation to cover testing and culling costs and testing of animals over 6 months of age prior to being introduced into the herd. We observe that in these departments, there was no significant increase in the annual prevalences. Other departments had more difficulties or acted as “buffer zones” for neighboring departments in highly affected regions (Cantal and Haute-Loire in particular). Our results confirm a significative increase in the annual prevalence in the department of Cantal (7.45% in 2021 and 10.61% in 2023). In Haute-de-Loire, the prevalence increased from 6.71 to 10.94% in 2022 and then decreased in 2023 to 6.34%. It is important to notice that data was only collected for the first 6 months and thus, the 2023% prevalences must be interpreted with caution as they might be underestimated The higher prevalence observed in Savoie, Haute-Savoie, Ain, Isère, and Puy-de-Dôme may be linked to the presence of extensive summer grazing areas in these departments, which are frequented by cattle from other regions potentially infected with *Besnoitia besnoiti* and may explain the higher prevalence found in these departments.

The lowest prevalence was 0.36% in the Rhône department in 2021, while the highest value was 23.44% in the Savoie department in 2021 (values calculated based on 97.5% and 100% of existing farms tested, respectively). In some departments and years, the proportion of farms tested was very low or even zero, which precluded estimating the corresponding prevalence. However, in all the departments, more than 96.2% of active farms were tested in at least one of the years of the study period (and in most of the departments, in all years). In 2023, the highest prevalence was observed in the departments of Isère and Cantal. However, these data should be interpreted with caution, as sample testing was only conducted up to July 1, 2023. Notably, *B. besnoiti* seroconversions in southern France have been shown to occur more frequently in spring (33), coinciding with increased activity of hematophagous insects during this period. As a result, the prevalence detected in bulk milk samples is likely to rise later in the season. The present study is one of the few using bulk milk samples to assess the prevalence of bovine besnoitiosis at farm level. Samples of bulk milk are easier to obtain and allow to more rapidly and inexpensively screen a large number of herds, compared to serological testing, which require the individual sampling of animals. Serological testing is, however, more adequate to determine animal and within-herd prevalence. Thus, one limitation of the study is the fact that prevalence in each department could only be determined at farm level. Previous studies carried out in dairy farms in Europe found within-herd seroprevalences of 59.8% in Portugal (28), 68% in Ireland (17) and 43.5% in Italy (30) and Grisez et al. (22) reported within-herd seroprevalences ranging between 42 and 92% in 8 dairy and beef farms in France. In an endemic area of Spain, the apparent seroprevalence in beef farms was 19.7% (38). In comparison, introduction of seropositive cattle into a naïve beef farm in Germany resulted in a within-herd animal seroprevalence between 89.4 and 100% over a 4-year longitudinal study (34). Other disadvantage of the present approach is that testing of bulk milk only reflects the status of cows being milked at the time of sampling. Dry cows, heifers, calves, males and cows whose milk is discarded (newly calved, cows with mastitis or undergoing treatment) were not included. Additionally, according to the manufacturer, the ELISA test used (ID Screen^®^ Besnoitia Milk Indirect ELISA) detects antibodies against *B. besnoiti* in bulk milk with a reported specificity of 100% (35). Regarding sensitivity, validation studies showed that the test detected antibodies in all herds with a high infection prevalence (>50%) and in 25% of herds with a prevalence between 3 and 7%, while none of the herds with a prevalence ≤ 1% tested positive. As such, our results may underestimate the true herd-level prevalence, as some farms in the sample may have had a low number of infected animals contributing to the bulk tank, falling below the detection threshold of the test. The highest prevalence was observed in departments that have summer grazing zones, where cattle from other departments are grouped together on pastures. The control of bovine besnoitiosis encompasses biosafety and biocontainment measures, as well as the serological testing of animals before they are introduced or re-introduced into a herd (22). On farms with < 10% prevalence, rapid culling of seropositive and clinically affected animals is the most effective approach. Culling of animals may not be economically feasible in farms with high prevalences. Here, separating animals into serologically positive and negative groups, avoiding their proximity, especially in the spring and summer months and preventing contact with animals from neighboring farms may help reducing infection along time. Situations of intermediate prevalence will have to be evaluated on a case-by-case basis (22). No vaccine is licensed in Europe. Live attenuated vaccines against bovine besnoitiosis based on tachyzoites grown in cell culture have been developed and are routinely used in Israel in stud bulls since 1986 (19). Although the vaccines protect animals from clinical disease, they do not prevent the introduction of infection into naïve herds. Moreover, vaccinated animals may still become subclinically infected and serve as reservoirs for parasite transmission (9). Several drugs appear promising in the fight against the parasite, but more studies are needed until an effective treatment against bovine besnoitiosis can be approved for marketing authorization (36).

## 5 Conclusion

The study aimed to characterize the presence of *B. besnoiti* infection in dairy farms in the Auvergne Rhône-Alpes region, France. The prevalence of the disease was estimated at the departmental level and its evolution assessed over the 4-year study period.

The prevalence of bovine besnoitiosis was highest in the departments of Savoie (17.83%), Haute-Loire (10.94%) and Isère (10.26%) in 2022. This might be related to the location of these departments, which feature wide grazing areas, and also act as a buffer zones for positive departments in other regions.

The annual prevalence increased significantly (*p* > 0.05, Table 10) in the departments of Cantal, Haute-Loire and Puy-de-Dome. Farm positivity was significantly associated with the number of animals on the farm (*p* < 0.05) and the number of tests conducted (*p* < 0.01, Table 6). Furthermore, mixed production farms appear to be more affected than dairy farms. Farms exhibited the lowest risk of testing positive in 2021 and the highest risk in 2023, supporting the need for targeted control measures.

In future research, it would be interesting to evaluate the seroprevalence of the disease at animal level, to determine within-herd prevalence. This information could be used to adjust control policies, such as test and cull programs, whose effectiveness is highly dependent on animal-level prevalence. This methodology could also be adapted to investigate beef cattle farms within the same region.

## Data Availability

The data analyzed in this study is subject to the following licenses/restrictions: the data are property of the Health Defense Group of the Auvergne Rhônes-Alpes region, France. Requests to access these data sets should be directed to Tiphaine Lahondes, lahondes.tiphaine@gmail.com.
